# Prevalence of inappropriateness of elemene injection for hospitalized cancer patients: a multicenter retrospective study

**DOI:** 10.3389/fphar.2024.1334701

**Published:** 2024-02-23

**Authors:** Mingzheng Cen, Guojun Jiang, Yuhua Zhao, Zhenwei Yu, Minxian Li

**Affiliations:** ^1^ The First Affiliated Hospital of Zhejiang Chinese Medical University (Zhejiang Provincial Hospital of Chinese Medicine), Hangzhou, China; ^2^ Affiliated Xiaoshan Hospital, Hangzhou Normal University, Hangzhou, China; ^3^ Sir Run Run Shaw Hospital, School of Medicine, Zhejiang University, Hangzhou, China

**Keywords:** appropriateness, elemene injection, cancer, chemotherapy, rational

## Abstract

**Background:** Elemene injection could provide clinical benefit for the treatment of various cancers, but the clinical evidence is weak. Thus, its wide use in China has raised concerns about the appropriateness of its use.

**Methods:** This was a multicenter retrospective study to evaluate the prevalence of inappropriateness of elemene injection for hospitalized cancer patients. Patients who met the inclusion criteria were retrospectively included, and demographic characteristics were extracted from the hospital information systems. The inappropriateness of elemene injection use was assessed using the preset criteria, and the prevalence was calculated. Multivariate logistic analysis was applied to identify any factors associated with inappropriate use.

**Results:** A total of 275 patients were included in the analysis. The median age was 62 years, and 30.9% were females. The most common cancer was lung cancer (24.0%), and 68.2% of the patients were receiving chemotherapy. The overall prevalence of inappropriateness was 61.8%. The most common reason for inappropriateness was inappropriate indications, and the second was inappropriate doses. Age and oncological department were significant risk factors associated with inappropriate use, while lung cancer, liver cancer and admission to cardiothoracic surgery were associated with a low risk of inappropriate use.

**Conclusion:** The prevalence of inappropriateness among hospitalized elemene injection users was high. More efforts, especially those to improve the appropriateness of indications, should be made to improve the rational use of elemene, as well as other complementary medicines. Physicians should take caution to avoid inappropriate use when prescribing drugs with limited clinical evidence.

## Introduction

Despite promising advances in cancer treatment in recent years, many challenges remain, such as drug resistance, metastasis, and severe adverse events associated with anticancer drugs (Ramos-Casals et al., 2020; Bagchi et al., 2021). People have tried to find new strategies to treat ethnodrugs, especially traditional Chinese medicines (Su et al., 2020). Elemene is the major active ingredient extracted from the rhizome of *Curcuma wenyujin* (Zhai et al., 2019). Its formulations, including oral emulsion and injection, were approved by the CFDA for the treatment of various cancers approximately 20 years ago (Bai et al., 2021). Elemene injection yields three isomers (δ, α, β), and β-elemene (1-methyl-1-vinyl-2,4-diisopropenyl-cyclohexane) is the predominant component. It has shown various antitumor effects in preclinical studies. Elemene can directly inhibit the proliferation and growth of various tumor cells; for example, it inhibits human cervical cancer cells in a concentration- and time-dependent manner, and the mechanism may be associated with the upregulation of P15 expression and the downregulation of cyclin D1 expression (Wang et al., 2018). A previous study also confirmed that elemene could induce apoptosis and exhibit antitumor effects (Liu et al., 2017). Other effects involved in the antitumor effect of elemene include the inhibition of tumor cell invasion and metastasis, reversal of multidrug resistance, enhancement of chemoradiotherapy sensitization, activation of protective autophagy, and regulation of the immune system (Xu et al., 2018; Qureshi et al., 2019; Tong et al., 2020; Bai et al., 2021; Tan et al., 2021). Many meta-analyses have also confirmed the benefit of elemene as a combined therapy or adjuvant therapy for the treatment of cancers (Wang et al., 2019; Liu et al., 2020). However, most of the included clinical studies were of low quality, and a recent umbrella review concluded that the benefits of elemene injection need to be proven by additional convincing trials. Moreover, no other regulatory agencies, such as the FDA or EMA, have approved the clinical use of elemene. Thus, we believe that the clinical evidence for elemene injection is weak, the benefits are uncertain, and elemene injection should be administered only to specific patients.

The use of complementary medicine, including elemene injection, is common in cancer patients and results in a substantial economic burden (Nie et al., 2023). This has raised concerns about the appropriateness of elemene use. Inappropriate use of drugs occasionally leads to the absence of clinical effects, but in most circumstances, adverse effects can occur, causing aggravation of the illness, additional diagnostic testing, and increased costs for the patient and health welfare system (Galimberti et al., 2022). Potential inappropriate drug use was significantly associated with a range of health-related and system-related outcomes (Mekonnen et al., 2021). The appropriateness of antibiotics, proton pump inhibitors, and some other drugs was assessed from different perspectives, and the results were unsatisfactory to various degrees (Khatter et al., 2021; Ardoino et al., 2022; Butler et al., 2022). Currently, there are few data regarding the appropriateness of elemene injection. These data are important for the improved application and management of elemene injection. We subsequently carried out this multicenter retrospective study to determine the prevalence of inappropriateness of elemene injection use in hospitalized patients with cancer.

## Materials and methods

### Study design and ethical approval

This was a multicenter retrospective study in which the prevalence of inappropriateness of elemene injection was evaluated in hospitalized cancer patients. The study was approved by the Ethics Committee of Sir Run Run Shaw Hospital, School of Medicine, Zhejiang University, with reference number 2023-0293. Informed consent was waived as part of the approval due to the retrospective nature of the study.

### Patient inclusion criteria

Patients were retrospectively searched in the hospital information system according to the following criteria: 1) had a diagnosis of cancer; 2) were admitted to Affiliated Xiaoshan Hospital, Hangzhou Normal University or Sir Run Run Shaw Hospital, School of Medicine, Zhejiang University; 3) were hospitalized from January 2021 to December 2021; and 4) received elemene injection treatment. The researchers reviewed the medical history, checked the eligibility of the patients and included eligible patients.

### Data collection

The following data were extracted from the hospital information system and medical history: age, sex, diagnosis, admission department, days of hospital stay, dose regimen of elemene injection, and combined therapy.

### Assessment of inappropriateness

The criteria for the inappropriateness of elemene injection use were set according to the drug label and clinical evidence. The detailed criteria were as follows: 1) Indication. The indications for elemene injection were limited to lung cancer, liver cancer, esophageal cancer, nasopharyngeal carcinoma, brain cancer, metastatic tumors of bone, gastric cancer, malignant pleural effusion and ascites. It would be inappropriate to use elemene for the treatment of other types of cancer. 2) Dosage and administration. Elemene injection should be administered intravenously at a dose of 352–528 mg every day. A dose that is not in the range is treated as inappropriate. For the treatment of malignant pleural effusion and ascites, these agents should be injected locally. The treatment duration should be no more than 21 days. 3) Contradiction. Patients with high fever or uncontrolled infection should not receive elemene. It is inappropriate to prescribe elemene injection to these patients. 4) Special patients. Patients who are pregnant or breastfeeding should be carefully evaluated for the risk and benefit of elemene use. 5) Caution. Patients with thrombocytopenia or bleeding risk should be carefully evaluated for the benefit and risk of elemene use. If no information about the evaluation was found in the patient’s medical history, it was considered inappropriate. The inappropriateness of each patient was assessed according to the inappropriateness criteria and personal medical history. If any criteria were met, it would be concluded that the elemene use in that patient was inappropriate.

### Statistical analysis

The overall prevalence of inappropriateness was calculated as the percentage of patients who did not fully meet the appropriate criteria for elemene injection. The patients were subsequently divided into two groups according to the appropriateness of the treatment. Univariate logistic analysis was performed first to test the difference in patient characteristics between groups, and any variables with a *p*-value less than 0.05 were subjected to stepwise multivariate logistic analysis, which eliminated any variables with a *p*-value larger than 0.05 step by step. The remaining variables in the multivariate logistic analysis were found to be independent factors associated with the appropriate use of elemene. The statistical analysis was performed using SPSS software.

## Results

### Patient inclusion

A total of 275 patients met the inclusion criteria and were included in the analysis. The patient demographic characteristics are shown in Table 1. Most of the patients were old. Various cancers were included, while most common was lung cancer. Elemene injection was combined with chemotherapy in the majority of patients. Notably, the median treatment length was 3 days.

**TABLE 1:** Demographic characteristics of the included patients.

Characteristic	Total
Age (years)	62 (55–67)
Sex	
Female	85 (30.9%)
Male	190 (69.1%)
Diagnosis	
Lung cancer	66 (24.0%)
Colorectal cancer	35 (12.7%)
Gastric cancer	30 (10.9%)
Liver cancer	28 (10.2%)
Pancreatic cancer	26 (9.45%)
Throat cancer	23 (8.36%)
Esophageal cancer	22 (8.00%)
Biliary tract cancer	16 (5.82%)
Genital tract tumors	10 (3.64%)
Other cancer	19 (6.90%)
Department	
General surgery	97 (35.3%)
Radiosurgery department	83 (30.2%)
Oncology department	58 (21.1%)
Cardiothoracic surgery	37 (13.5%)
Length of hospital stay (days)	7 (4–17)
Dosing regimen of Elemene	
Dose (mg)	440 (264–528)
Duration of therapy (days)	3 (2–5)
Combined treatment	
Chemotherapy	199 (68.2%)
Radiotherapy	28 (9.58%))
Surgery	21 (6.84%)
Immunotherapy	15 (5.45%)
No treatment	45 (15.4%)

### Prevalence of inappropriateness of elemene injection use

**FIGURE 1:**
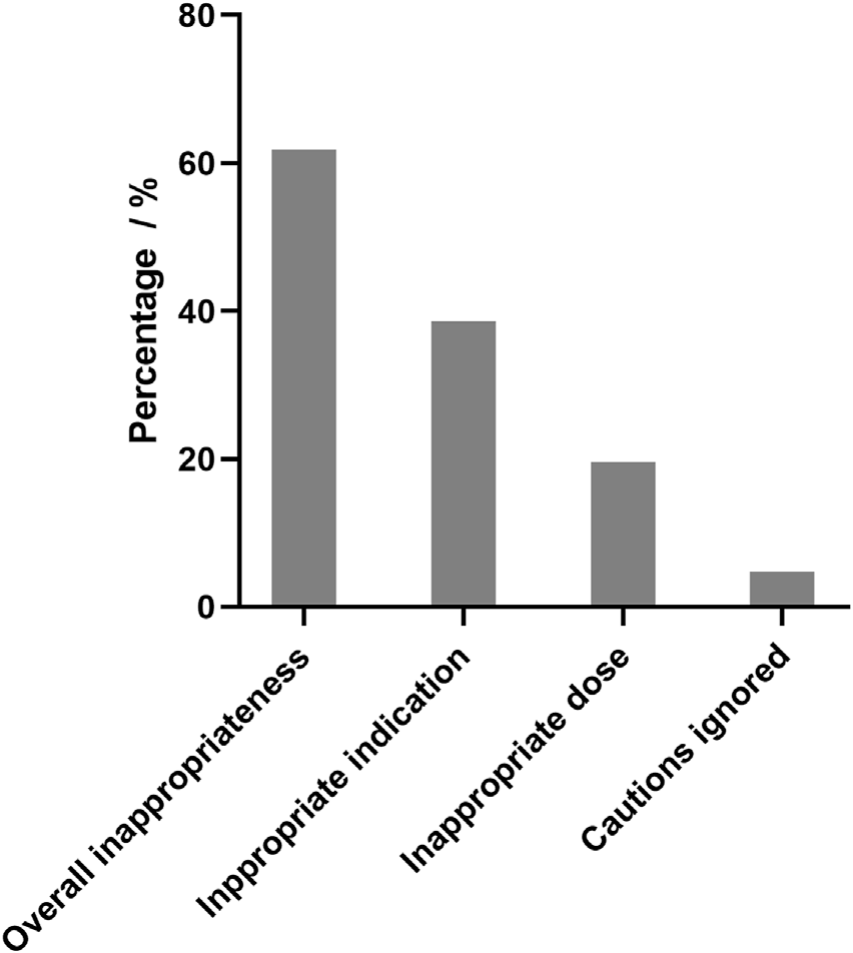
Overall prevalence of inappropriateness of elemene injection use in hospitalized patients with cancer. Appropriate indication means elemene injection can only be used for approved types of cancers, which are limited to lung cancer, liver cancer, esophageal cancer, nasopharyngeal carcinoma, brain cancer, metastatic tumors of bone, gastric cancer, malignant pleural effusion and ascites. Appropriate dose means elemene injection is administered intravenously at a dose of 352–528 mg every day. Caution ignored means elemene injection were used to patient with risk without evaluation. Patients with thrombocytopenia or bleeding risk should be carefully evaluated for the benefit and risk of elemene use.

As shown in Figure 1, the overall prevalence of inappropriate elemene injection use was 61.8%. The most common cause of inappropriate use is inappropriate indications. Many types of cancer, such as colorectal cancer and pancreatic cancer, have not been approved for treatment, and it is inappropriate to use elemene in these patients. The personal characteristics of appropriate use and inappropriate use are shown in Table 2. According to the results of the multivariate analysis, age and oncological department status were significant risk factors associated with inappropriate use, while lung cancer, liver cancer and admission to a cardiothoracic surgery were associated with a low risk of inappropriate use.

**TABLE 2:** Demographics of the patients in each group and logistic regression analysis.

Variable	Appropriate (n = 105)	Inappropriate (n = 170)	*p*-Value^†^	*p*-Value^‡^	OR^‡^
Age (years)	58 (51–65)	63 (56–67)	0.001	0.028	0.96 (0.93–1)
Sex					
Female	23 (21.9)	62 (36.47)	0.011	0.224	
Male	82 (78.1)	108 (63.53)	-		
Diagnosis*					
Lung cancer	44 (41.9)	22 (12.94)	<0.001	0.001	4.71 (1.91–11.58)
Gastric cancer	12 (11.43)	18 (10.59)	0.828		
Liver caner	22 (20.95)	6 (3.53)	<0.001	<0.001	12.02 (4.31–33.55)
Throat cancer	13 (12.38)	10 (5.88)	0.064		
Esophageal cancer	12 (11.43)	10 (5.88)	0.105		
Colorectal cancer	0	35 (20.6)	-		
Pancreatic cancer	0	26 (15.3)	-		
Biliary tract cancer	0	16 (9.41)	-		
Genital tract cancer	0	10 (5.88)	-		
Other	2 (1.90)	17 (10.0)	-		
Department					
General surgery	33 (31.43)	64 (37.65)	0.295		
Radiosurgery department	34 (32.38)	49 (28.82)	0.533		
Oncology department	3 (2.86)	55 (32.35)	<0.001	0.001	0.12 (0.03–0.43)
Cardiothoracic surgery	35 (33.33)	2 (1.18)	<0.001	<0.001	29.37 (6.24–138.18)
Length of stay (days)	6 (4–10)	10 (4–24)	<0.001	0.188	
Duration of therapy (days)	3 (2–4)	3 (2–6)	0.055		
Combined treatment					
Chemotherapy	85 (80.95)	114 (67.06)	0.017	0.976	
Radiotherapy	6 (5.71)	22 (12.94)	0.061		
Surgery	9 (8.57)	12 (7.06)	0.647		
Immunotherapy	12 (11.43)	3 (1.76)	0.008	0.004	6.94 (1.67–28.84)
No combined treatment	10 (9.52)	35 (20.59)	0.019	0.879	

^†^ *p*-value of univariate logistic analysis; ^‡^ *p*-value and odds ratio of multivariate logistic analysis.

*Statistical analysis was not carried out for unapproved indications of elemene injection.

## Discussion

To the best of our knowledge, this is the first study to evaluate the prevalence of inappropriateness of elemene injection in hospitalized cancer patients. Surprisingly, the overall inappropriateness rate was high. Only 38.2% of the patients received elemene injection appropriately, and the main reason for inappropriate use was inappropriate indications. Our results highlight the need to pay attention to the rational use of elemene injection, and efforts should be made to reduce inappropriate use.

The prevalence of inappropriateness was higher than expected. This raised concerns about its rational use, as well as other complementary medicines. Although the outcome of inappropriate use of elemene injection was not evaluated in this study, previous studies had proved that inappropriate medicine use in cancer patients always associated with high risk of adverse effects and unfavorite outcome of therapy (Krečak et al., 2023; Mohamed et al., 2023). The reason for the prevalent inappropriate use of elemene injection might be as follows. First, physicians do not always care about the indications for complementary medicine, including elemene injection. Although elemene has various antitumor effects, its approved indications are limited. Physicians should be informed that elemene is not suitable for all types of cancer. Second, similar to other medicines, marketing efforts can increase the unnecessary use of elemene injection and increase the overall prevalence of inappropriateness (Yu et al., 2020). The healthcare system should also be aware of this effect. Finally, patients in East Asia have expectations for complementary medicine and would like to receive these medicines voluntarily (Sun et al., 2018).

The main cause of inappropriateness was inappropriate indication, and colorectal cancer was the most common nonindication use of elemene. The effect of elemene on colorectal cancer has been supported by preclinical studies, but clinical evidence for this cancer is rare (Chen et al., 2020; Wang et al., 2022). It is unclear whether patients with colorectal cancer could benefit from elemene treatment. Other nonindication uses of elemene, such as in pancreatic carcinoma and biliary tract cancer, have only been investigated in in vitro studies (Long et al., 2019; Wu et al., 2022). These findings indicated that efforts to reduce elemene use in patients without suitable indications should be made preferentially. Other reasons for inappropriateness were the inappropriate dose and administration to cautious patients without evaluation. Patients sometimes receive elemene at a dose lower than suggested, and this should be avoided because no evidence is available. Despite the good safety of elemene in the treatment of cancers, it can also cause severe adverse effects (Gao et al., 2018). Adverse effects more easily occur in patients under physio-pathological conditions. Patients with thrombocytopenia should be carefully evaluated when dosing elemene. Unfortunately, it is overlooked in clinical practice according to the results of our study.

The appropriateness of these treatments is significantly greater in patients with lung cancer and liver cancer. This may be associated with additional experience using elemene injection for treating these cancers. As mentioned above, lung cancer and liver cancer are approved indications of elemene injection. Numerous clinical studies have been carried out to assess the efficacy of elemene in treating lung cancer and liver cancer in combination with chemotherapy or radiotherapy (Jiang et al., 2017; Yao et al., 2019; Yang et al., 2022). The appropriateness of elemene use differed greatly among departments. Interestingly, admission to the oncological department was associated with a high risk of inappropriate use, but admission to the cardiothoracic surgery department was associated with a low risk of inappropriate use. Physicians in the oncology department specialize in cancer treatment, but they fail to appropriately use elemene injection. The reason for better appropriateness of surgery in the cardiothoracic surgery department is that lung cancer, the most common indication for elemene injection, is the main cancer type in this department.

Notably, older age is an independent risk factor for inappropriate use of elemene injection. Thus, more attention should be given to these patients, as older patients more easily develop adverse drug events, especially when inappropriate drugs are used (Yao et al., 2019).

This study has several limitations. The included centers were limited. The effect of the appropriateness of elemene use on clinical outcome was not investigated in the present study. Moreover, bias may exist due to the retrospective nature of the study.

## Conclusion

This study assessed the prevalence of inappropriateness of elemene injection use in hospitalized patients with cancer. The results indicated that the overall prevalence of inappropriateness was as high as 61.8%. The main reason for inappropriateness was inappropriate indications. Moreover, several independent factors associated with inappropriate use were identified. This study raised the concern of the inappropriateness of elemene injection, as well as other complementary medicines. More efforts should be made to understand the status and improve the appropriate use of elemene injection. Physicians should make carefully evaluation and follow the guidance of inserts when prescribing drugs with limited clinical evidence, such as elemene injection, to avoid inappropriate use.

## Data Availability

The original contributions presented in the study are included in the article/Supplementary material, further inquiries can be directed to the corresponding authors.

## References

[B1] Ardoino I. Casula M. Molari G. Mucherino S. Orlando V. Menditto E. (2022). Prescription appropriateness of drugs for peptic ulcer and gastro-esophageal reflux disease: baseline assessment in the LAPTOP-PPI cluster randomized trial. Front. Pharmacol. 13, 803809. 10.3389/fphar.2022.803809 35418868 PMC8996306

[B2] Bagchi S. Yuan R. Engleman E. G. (2021). Immune checkpoint inhibitors for the treatment of cancer: clinical impact and mechanisms of response and resistance. Annu. Rev. Pathol. Mech. Dis. 16, 223–249. 10.1146/annurev-pathol-042020-042741 33197221

[B3] Bai Z. Yao C. Zhu J. Xie Y. Ye X. Y. Bai R. (2021). Anti-tumor drug discovery based on natural product β-elemene: anti-tumor mechanisms and structural modification. Molecules 26, 1499. 10.3390/molecules26061499 33801899 PMC7998186

[B4] Butler A. M. Brown D. S. Durkin M. J. Sahrmann J. M. Nickel K. B. O'Neil C. A. (2022). Erratum: association of inappropriate outpatient pediatric antibiotic prescriptions with adverse drug events and health care expenditures. JAMA Netw. Open 5, 2214153. 10.1001/jamanetworkopen.2022.14153 PMC9136626 35616940

[B5] Chen P. Li X. Zhang R. Liu S. Xiang Y. Zhang M. (2020). Combinative treatment of β-elemene and cetuximab is sensitive to KRAS mutant colorectal cancer cells by inducing ferroptosis and inhibiting epithelial-mesenchymal transformation. Theranostics 10, 5107–5119. 10.7150/thno.44705 32308771 PMC7163451

[B6] Galimberti F. Olmastroni E. Casula M. Merlo I. Franchi M. Catapano A. L. (2022). Evaluation of factors associated with appropriate drug prescription and effectiveness of informative and educational interventions—the EDU.RE.DRUG Project. Front. Pharmacol. 13, 832169. 10.3389/fphar.2022.832169 35548361 PMC9081494

[B7] Gao F. Shao Y. Zhong D. S. Liu X. Meng F. L. (2018). Severe adverse reactions induced by the chest injection of elemene: an analysis of 7 cases. Chin. J. Lung Cancer 21, 458–462. 10.3779/j.issn.1009-3419.2018.06.06 PMC6022031 29945704

[B8] Jiang X. Hidru T. H. Zhang Z. Bai Y. Kong L. Li X. (2017). Evidence of elemene injection combined radiotherapy in lung cancer treatment among patients with brain metastases: a systematic review and meta-analysis. Med. (United States) 96, e6963. 10.1097/MD.0000000000006963 PMC5457871 28538391

[B9] Khatter A. Moriarty F. Ashworth M. Durbaba S. Redmond P. (2021). Prevalence and predictors of potentially inappropriate prescribing in middle-aged adults: a repeated cross-sectional study. Br. J. Gen. Pract. 71, E491–E497. 10.3399/BJGP.2020.1048 33606659 PMC8136579

[B10] Krečak I. Pivac L. Lucijanić M. Skelin M. (2023). Polypharmacy, potentially inappropriate medications, and drug-to-drug interactions in patients with chronic myeloproliferative neoplasms. Biomedicines 11, 1301. 10.3390/biomedicines11051301 37238972 PMC10215953

[B11] Liu Y. Chen L. Zhang R. Chen B. Xiang Y. Zhang M. (2020). Efficacy and safety of elemene combined with chemotherapy in advanced gastric cancer: a Meta-analysis. Med. (United States) 99, E19481. 10.1097/MD.0000000000019481 PMC7220410 32176081

[B12] Liu Y. Jiang Z. Y. Zhou Y. L. Qiu H. hui, Wang G. Luo Y. (2017). β-elemene regulates endoplasmic reticulum stress to induce the apoptosis of NSCLC cells through PERK/IRE1α/ATF6 pathway. Biomed. Pharmacother. 93, 490–497. 10.1016/j.biopha.2017.06.073 28672279

[B13] Long J. Liu Z. Hui L. (2019). Anti-tumor effect and mechanistic study of elemene on pancreatic carcinoma. BMC Complement. Altern. Med. 19, 133. 10.1186/s12906-019-2544-2 31215421 PMC6582541

[B14] Mekonnen A. B. Redley B. de Courten B. Manias E. (2021). Potentially inappropriate prescribing and its associations with health-related and system-related outcomes in hospitalised older adults: a systematic review and meta-analysis. Br. J. Clin. Pharmacol. 87, 4150–4172. 10.1111/bcp.14870 34008195 PMC8597090

[B15] Mohamed M. R. Mohile S. G. Juba K. M. Awad H. Wells M. Loh K. P. (2023). Association of polypharmacy and potential drug-drug interactions with adverse treatment outcomes in older adults with advanced cancer. Cancer 129, 1096–1104. 10.1002/cncr.34642 36692475 PMC10958985

[B16] Nie H. Han Z. Nicholas S. Maitland E. Huang Z. Chen S. (2023). Costs of traditional Chinese medicine treatment for inpatients with lung cancer in China: a national study. BMC Complement. Med. Ther. 23, 5. 10.1186/s12906-022-03819-3 36624405 PMC9827714

[B17] Qureshi M. Z. Attar R. Romero M. A. Sabitaliyevich U. Y. Nurmurzayevich S. B. Ozturk O. (2019). Regulation of signaling pathways by β-elemene in cancer progression and metastasis. J. Cell. Biochem. 120, 12091–12100. 10.1002/jcb.28624 30912190

[B18] Ramos-Casals M. Brahmer J. R. Callahan M. K. Flores-Chávez A. Keegan N. Khamashta M. A. (2020). Immune-related adverse events of checkpoint inhibitors. Nat. Rev. Dis. Prim. 6, 38. 10.1038/s41572-020-0160-6 32382051 PMC9728094

[B19] Su X. L. Wang J. W. Che H. Wang C. F. Jiang H. Lei X. (2020). Clinical application and mechanism of traditional Chinese medicine in treatment of lung cancer. Chin. Med. J. Engl. 133, 2987–2997. 10.1097/CM9.0000000000001141 33065603 PMC7752681

[B20] Sun L. Mao J. J. Vertosick E. Seluzicki C. Yang Y. (2018). Evaluating cancer patients’ expectations and barriers toward traditional Chinese medicine utilization in China: a patient-support group–based cross-sectional survey. Integr. Cancer Ther. 17, 885–893. 10.1177/1534735418777117 29888609 PMC6142069

[B21] Tan T. Li J. Luo R. Wang R. Yin L. Liu M. (2021). Recent advances in understanding the mechanisms of elemene in reversing drug resistance in tumor cells: a review. Molecules 26, 5792. 10.3390/molecules26195792 34641334 PMC8510449

[B22] Tong H. Liu Y. Jiang L. Wang J. (2020). Multi-targeting by β-elemene and its anticancer properties: a good choice for oncotherapy and radiochemotherapy sensitization. Nutr. Cancer 72, 554–567. 10.1080/01635581.2019.1648694 31387393

[B23] Wang G. Y. Zhang L. Geng Y. Wang B. Feng X. J. Chen Z. L. (2022). β-Elemene induces apoptosis and autophagy in colorectal cancer cells through regulating the ROS/AMPK/mTOR pathway. Chin. J. Nat. Med. 20, 9–21. 10.1016/S1875-5364(21)60118-8 35101253

[B24] Wang L. Zhao Y. Wu Q. Guan Y. Wu X. (2018). Therapeutic effects of β-elemene via attenuation of the Wnt/β-catenin signaling pathway in cervical cancer cells. Mol. Med. Rep. 17, 4299–4306. 10.3892/mmr.2018.8455 29363722 PMC5802201

[B25] Wang X. Liu Z. Sui X. Wu Q. Wang J. Xu C. (2019). Elemene injection as adjunctive treatment to platinum-based chemotherapy in patients with stage III/IV non-small cell lung cancer: a meta-analysis following the PRISMA guidelines. Phytomedicine 59, 152787. 10.1016/j.phymed.2018.12.010 31005810

[B26] Wu Q. Shi X. Pan Y. Liao X. Xu J. Gu X. (2022). The chemopreventive role of β-elemene in cholangiocarcinoma by restoring PCDH9 expression. Front. Oncol. 12, 874457. 10.3389/fonc.2022.874457 35903688 PMC9314746

[B27] Xu L. Guo T. Qu X. Hu X. Zhang Y. Che X. (2018). β-elemene increases the sensitivity of gastric cancer cells to TRAIL by promoting the formation of DISC in lipid rafts. Cell Biol. Int. 42, 1377–1385. 10.1002/cbin.11023 29957841

[B28] Yang S. Zheng L. Sun Y. Li Z. (2022). Effect of network-based positive psychological nursing model combined with elemene injection on negative emotions, immune function and quality of life in lung cancer patients undergoing chemotherapy in the era of big data. Front. Public Heal. 10, 897535. 10.3389/fpubh.2022.897535 PMC9120652 35602129

[B29] Yao Y. Chen J. Jiao D. Li Y. Zhou X. Han X. (2019). Elemene injection combined with transcatheter arterial chemoembolization for unresectable hepatocellular carcinoma: a meta-analysis. Med. Baltim. 98, e17813. 10.1097/MD.0000000000017813 PMC6946248 31689867

[B30] Yu Z. Zhang J. Zheng Y. Yu L. (2020). Trends in antidepressant use and expenditure in six major cities in China from 2013 to 2018. Front. Psychiatry 11, 551. 10.3389/fpsyt.2020.00551 32765307 PMC7378967

[B31] Zhai B. Zhang N. Han X. Li Q. Zhang M. Chen X. (2019). Molecular targets of β-elemene, a herbal extract used in traditional Chinese medicine, and its potential role in cancer therapy: a review. Biomed. Pharmacother. 114, 108812. 10.1016/j.biopha.2019.108812 30965237

